# Transfer Entropy Analysis of Interactions between Bats Using Position and Echolocation Data

**DOI:** 10.3390/e22101176

**Published:** 2020-10-19

**Authors:** Irena Shaffer, Nicole Abaid

**Affiliations:** 1 Engineering Mechanics Program, Virginia Tech, Blacksburg, VA 24061, USA; irenas4@vt.edu; 2 Department of Mathematics, Virginia Tech, Blacksburg, VA 24061, USA

**Keywords:** transfer entropy, bat swarms, animal group interaction, 3D tracking, microphone arrays

## Abstract

Many animal species, including many species of bats, exhibit collective behavior where groups of individuals coordinate their motion. Bats are unique among these animals in that they use the active sensing mechanism of echolocation as their primary means of navigation. Due to their use of echolocation in large groups, bats run the risk of signal interference from sonar jamming. However, several species of bats have developed strategies to prevent interference, which may lead to different behavior when flying with conspecifics than when flying alone. This study seeks to explore the role of this acoustic sensing on the behavior of bat pairs flying together. Field data from a maternity colony of gray bats *(Myotis grisescens)* were collected using an array of cameras and microphones. These data were analyzed using the information theoretic measure of transfer entropy in order to quantify the interaction between pairs of bats and to determine the effect echolocation calls have on this interaction. This study expands on previous work that only computed information theoretic measures on the 3D position of bats without echolocation calls or that looked at the echolocation calls without using information theoretic analyses. Results show that there is evidence of information transfer between bats flying in pairs when time series for the speed of the bats and their turning behavior are used in the analysis. Unidirectional information transfer was found in some subsets of the data which could be evidence of a leader–follower interaction.

## 1. Introduction

Many social animals such as insects, fish, birds, and bats exhibit collective behavior, where one individual adjusts its behavior in response to other members of the group [1,2]. One aspect of collective behavior that has received much interest is collective motion of animal groups [3,4,5], which results in impressive displays by coordinated bird flocks and fish schools for example, and has applications in engineering multi-agent or distributed systems. While these behaviors have long been studied from the point of view of mathematical models, one relatively new method of studying collective motion is information theory [3].

Information theoretic tools such as mutual information, causation entropy, and transfer entropy have recently become popular methods for studying the interactions of individuals in a model-free context. Information theory is based on work by Shannon [6] and has been used in many studies on topics such as computational neuroscience [7,8,9], analysis of complex systems [10], collective robotics [11,12], weather and climate science [13], and social media [14,15]. Specifically, transfer entropy, an extension of Shannon’s concept of entropy to measure information flow between time series, has been used to study the interaction of animal groups such as fish [16], meerkats *(Suricat suricatta)* [17], and insects [18]. It has also previously been used to determine leader–follower interactions in meerkats [17], bats [19,20], and in fish–robot interactions [21].

Previous studies have used information theory to model the interaction between bats flying in pairs [19,20]. Using 3D trajectories extracted from videos of wild bats in flight, these studies used transfer entropy and convergent cross mapping to identify interaction between bats. The results from the studies show evidence of information exchange between bats in a pair as well as greater information flow from bats flying behind their partners in space, indicating that the rear bat is leading the pair.

Echolocating bats are somewhat unique from other social animals in that they use the active sensing mechanism of echolocation as their primary means of navigation and obstacle avoidance [22]. Bats emit directional ultrasonic pulses and are able to receive information about their environment based on the spectral changes, amplitude, and time delay of the returning echoes [23]. Since the bats must be able to compare the echo to the original call, they risk interference from calls made by nearby conspecifics, also known as sonar jamming [24]. Many bat species are social and live in colonies ranging in size from tens to millions of bats for at least part of their annual life-cycle [25,26]. When roosting in such colonies, they often emerge from roosts nightly in large swarms and fly in groups at high speeds. Since these bats rarely collide with each other or objects in their environment, it suggests they have developed strategies to avoid signal interference. Studies supporting this idea have shown that some of the sensing strategies used by bats may be a response to sonar jamming, such as frequency modulation [27,28,29], adjusting pulse emission rates [30,31,32], and eavesdropping on conspecifics [33,34,35].

One strategy used by bats to prevent sonar jamming is adjusting pulse emission rates. In one study, wild-caught Mexican free-tailed bats (*Tadarida brasiliensis Mexicana*) were placed in a wire cage in a recording studio, and the number of calls made by the bats were counted [30]. It was found that artificial calls played in the recording studio caused slower emission rates by the bats, and this reduced the relative proportion of emitted pulses that overlapped with another bat. In another study, Mexican free-tailed bats were recorded flying in a laboratory where artificial calls were directed at varying angles to the bat’s flight trajectory [31]. The bats reduced their call emission rates in the presence of the artificial calls and had the worst navigational performance when the artificial calls where produced from behind the bats in the direction they were flying, leading to the conclusion that the direction of a conspecific’s call plays a role in the amount of interference. It is worth noting that these responses may depend heavily on species. For example, observations of Eastern bent-wing bats *(Miniopterus fuliginosus)* exiting a cave, suggested this species increased their call emission rate when more bats were present [32].

As a more extreme response to sonar jamming, bats will sometimes cease making calls for a short time and instead listen to the pulses produced by another bat. Chiu et al. recorded pairs of big brown bat *(Eptesicus fuscus)* in a laboratory setting and found that when the bats were flying close together, one would often fly silently for more than 0.2 s [33]. Since it has been shown that bats can passively locate sources of sound, though with less accuracy than through active sensing [34], this study suggests that the silent bats were able to use the calls made by the conspecific to avoid collisions. Another study of hoary bats *(Lasiurus cinereus)* in the wild also found some cases where these bats flew in silence for a short time [35].

Based on this evidence, this study seeks to test the hypothesis that bats listen to the calls of their conspecifics. More specifically, this leads to a question that has not received much attention: how much of a role do sonar calls play in the interaction of flying bats? The aim of this study was to use transfer entropy to analyze the interactions of pairs of bats in flight and to expand upon and clarify previous work which uses 3D trajectories by incorporating the calls made by each bat into the analysis. While previous works have studied the role of echolocation calls in bat interactions, to the best of our knowledge none of these have used information theoretic techniques to measure this interaction. We believe that using these techniques gives additional insight into the behavior of bats in flight.

## 2. Materials and Methods

### 2.1. Experimental Setup and Data Collection

**Figure 1:**
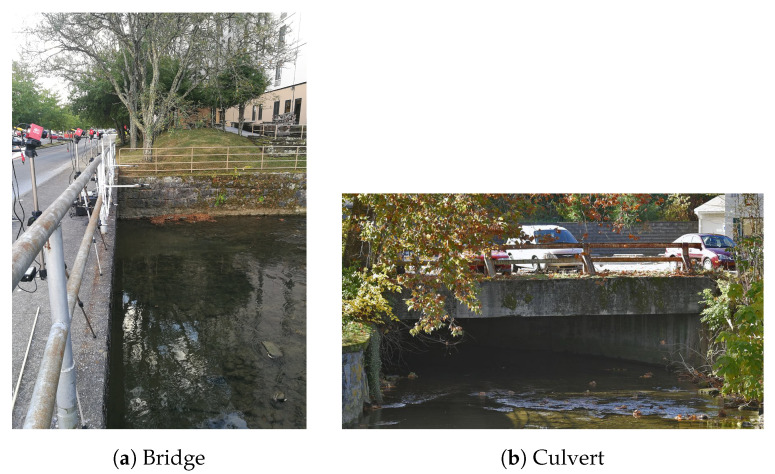
Pictures of the bridge over Beaver Creek with the cameras and microphones set up for recording and the culvert where the bats roost. Culvert photo credit [36]. Photo of the bridge was taken 12 September 2019.

Data for this study were collected in September 2019 from a wild bat colony in Bristol, VA, USA. A maternity colony of about 10,000 gray bats *(Myotis grisescens)* roosts in a culvert, diverting Beaver Creek, that runs under Bristol (Figure 1). At sunset, the bats emerge from the culvert and fly along the creek to forage for insects. Four thermal cameras and an array of eight microphones were placed on a bridge a short distance from the opening of the culvert to record the bats as they flew along the creek and under the bridge. We note that the data collected here were not used in previous studies.

#### 2.1.1. Cameras

**Figure 2:**
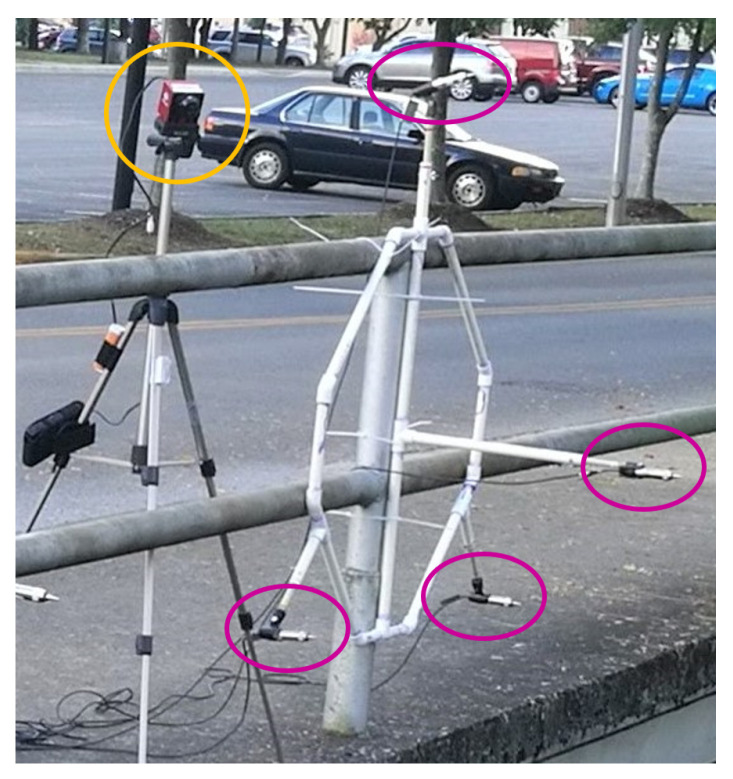
Thermal camera (in yellow circle) and one array of microphones (in magenta ovals).

Four thermal cameras (Viento 640, Sierra Olympic, Hood River, OR, USA) with video acquisition rate of 30 Hz, were used to film the bats’ flight paths (Figure 2). The cameras were placed in a line with approximately 1 m between each camera. All the cameras were set at different angles to focus on a point about 5 m from the bridge. Using the four views from the cameras, it is possible to obtain 3D positions of points in a volume of approximately 5 m × 5 m × 8 m with the longest dimension aligned with the width of the creek.

To calibrate the camera system for stereoscopic tracking [37], the intrinsic parameters of each camera were found by filming a checkerboard with squares of known size and using MATLAB’s Camera Calibration Toolbox [38]. To find the extrinsic parameters of the cameras, a wand with a known length was moved though the space seen by all the cameras. The ends of the wand were heated to be easily seen by the thermal cameras. This was done when all cameras had been set up in position to record the bats, but before any bats had exited the culvert. The ends of the wand were tracked using a custom digitizing software, DLT [39]. The extrinsic parameters were calculated using the easyWand software [40], which relies on open source sparse bundle adjustment routines [41].

**Figure 3:**
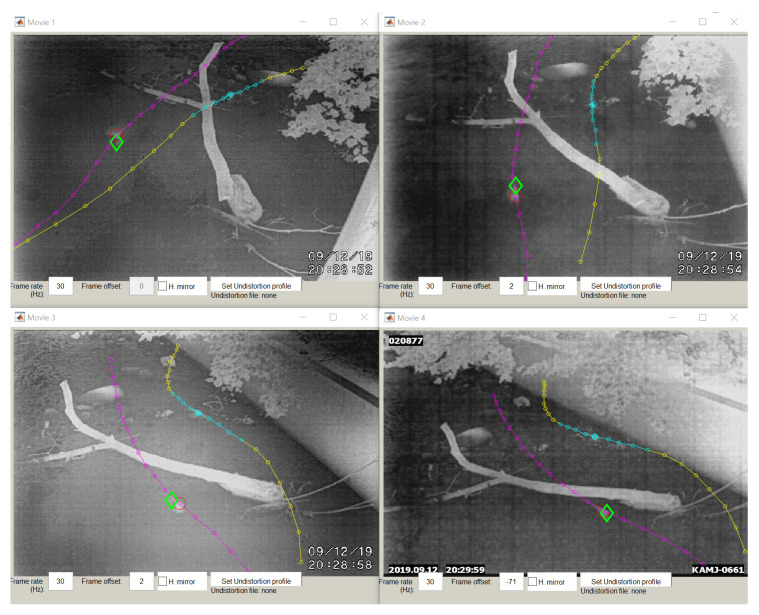
Flight path of a pair of bats projected into all four camera views as tracked using the DLT interface [39]. Tracked points are shown in magenta, cyan, and yellow.

**Figure 4:**
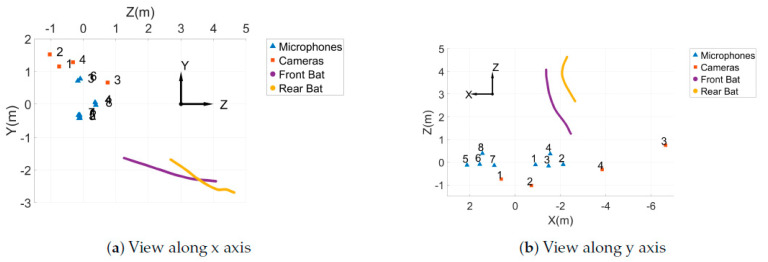
3D trajectories of the bat pair shown in Figure 3, where (**a**) gives the view along the x axis and (**b**) gives the view along the y axis. The positions of the cameras and microphones are also shown.

The cameras started recording when the bats began to leave the culvert, around sunset, and were stopped only after most bats had exited, which took approximately 45 min. Pairs of bats were tracked by hand from the videos to obtain their 3D trajectories. The tracking and acquisition of 3D points were done in the same digitizing software used to track the wand points [39]. Figure 3 shows a pair of bats that have been tracked in all four camera views. Pairs were defined as two bats which could both be seen by all cameras for at least 10 frames while no other bats were in view of the cameras. The first bat to enter the view of the cameras was designated the front bat and the second, the rear bat. The 3D trajectories were smoothed using the MATLAB smooth function with the lowess method, a local regression using weighted linear least squares and a 1st degree polynomial model [42], and then interpolated using a cubic spline and re-sampled at 120 Hz. Only the trajectories at times in which both bats were in view of the cameras were used for analysis. An example of the 3D trajectory of a pair of bats can be seen in Figure 4.

#### 2.1.2. Microphone Array

**Figure 5:**
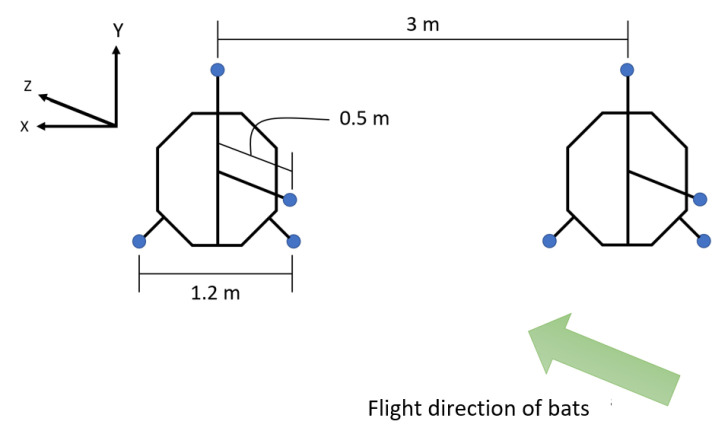
Placement of microphones in the array, where blue circles represent the microphones.

An array of eight microphones (USG Omidirectional Electret Ultrasound Microphones, Knowles FG-O, Avisoft Bioacoustics, Nordbahn, Germany) with an acquisition rate of 250 kHz and a recording interface (UltraSoundGate 816H, Avisoft Bioacoustics, Nordban, Germany) were used in this study to record sounds made by the bats (Figure 2). The microphones were placed in two star arrays about 3 m apart. The positions of the microphones in the array can be seen in Figure 5.

**Figure 6:**
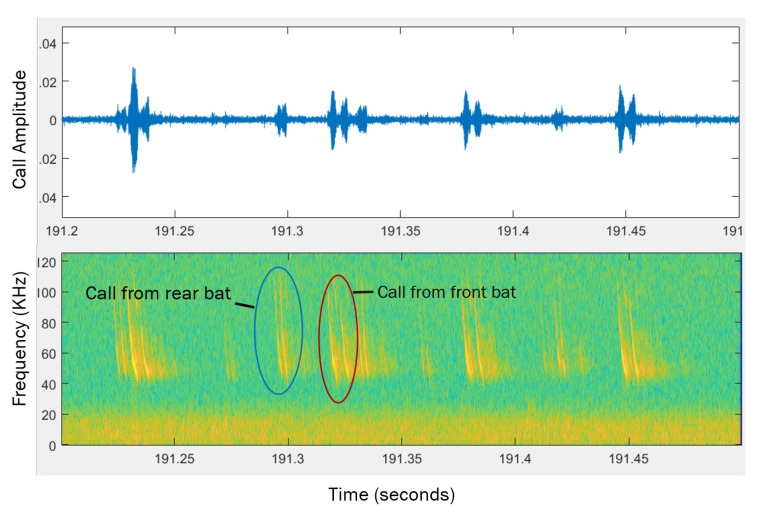
An example of calls recorded by the microphones. Top plot shows the microphone voltage produced by the calls, lower plot shows a spectrogram of the calls. Two calls have been identified as being produced by the front and rear bat.

When recording in the field, the microphones were pointed horizontally in direction of the negative z axis as shown in Figure 4 so they were aimed in the direction the bats were coming from. The set up of one of the thermal cameras and one of the microphone arrays can be seen in Figure 2. In order to synchronize the time on the videos and audio recordings, two metal objects were hit together in view of one of the cameras. This produced a sound that was picked up by the microphones at virtually the same time the impact can be seen in the video. After selecting bat pairs from the videos, the calls made by the bats were analyzed using Batalef [43], a custom software program for sound analysis with special features specifically designed for identifying and analyzing bat calls. After finding all the calls made by a pair of bats, each call was manualy assigned to the front or rear bat by looking at the amplitude and differences in the spectrogram of the call. These call assignments were verified using MATLAB’s k-means cluster classification. An example of the spectrogram of some calls can be seen in Figure 6.

### 2.2. Data Analysis

Transfer entropy (TE) is the measure of information flow from one time series to another and was developed by Schreiber [44]. The TE is based on the Shannon entropy which is the expected uncertainty in a random variable [6]. Shannon entropy is defined as:

(1) $$H\left(X\right)=-\sum_{x\in X}^{}p\left[x\right]\log_{2}p\left[x\right],$$

where *x* is a measurement of a random variable *X*, X is the set containing all possible values of *X*, and p[x] is the probability distribution function (PDF) of *X*.

Transfer entropy, $T_{X\to Y}$, measures the amount of information that flows from *X* to *Y*. It is calculated as the amount of information the source, x(t), provides about the next state of the destination, y(t+1), in relation to the current state, y(t). It is defined as:

(2) $$T_{X\to Y}=\sum_{\begin{array}{c} y(t+1)\in Y(t+1)\\ y(t)\in Y(t)\\ x(t)\in X(t) \end{array}}p\left[y\left(t+1\right),y\left(t\right),x\left(t\right)\right]\log\frac{p[y(t+1)|y(t),x(t)]}{p[y(t+1)|y(t)]},$$

where p[y(t+1),y(t),x(t)] is the joint probability of y(t+1),y(t), and x(t); p[y(t+1)|y(t),x(t)] is the probability of y(t+1) conditioned on y(t) and x(t); and p[y(t+1)|y(t)] is the probability of y(t+1) conditioned on y(t). For discrete time series data, a logarithm base of 2 is used and the units of TE are bits. For continuous data, a logarithm base of e is used and the units of TE are nats [45]. Transfer entropy is asymmetric so, in general, $T_{X\to Y}\neq T_{Y\to X}$. Due to this, the direction of information flow in a system can be determined.

There are a variety of different methods that can be used to construct the PDF used to calculate the TE [45]. One method is to sort the data into a finite number of bins and count the number of data points in each bin to determine the probability function. While this method is very fast, it is not as accurate as other methods. More complex and accurate methods include: a multivariate Gaussian model, kernel estimation, permutation entropy, and the Kraskov, Stögbauer, and Grassberger (KSG) technique [46]. Kernel estimation measures the similarity between pairs using a specific resolution. The KSG technique improves on the kernel estimation method by using a dynamically altered kernel width that adjusts with sample density. The KSG method can measure non-linear relationships, is model free, and requires fewer observations to achieve an accurate PDF estimations. While originally used to calculate mutual information, it has been extended to also calculate transfer entropy [47]. For these reasons, the KSG technique is one of the most widely used PDF estimators [45]; we use it for this study.

**Figure 7:**
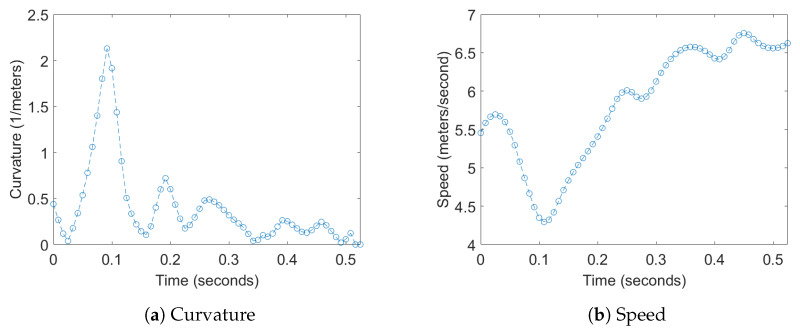
Examples of plots the curvature of the trajectory, and speed of a single bat.

Transfer entropy was computed for two different 1D time series derived from the positions of the bats; this analysis was then extended to a multidimensional version by augmenting these time series with the acoustic data captured by the microphone array, which is detailed later. For the position data, one of time series used was curvature of the flight trajectories which was chosen to measure the response of one bat to a change in direction of another bat. The second time series used was the flight speed, which was chosen to measure the response of one bat to a change in the velocity of another bat. Examples of these 1D time series for a bat can be seen in Figure 7.

The 3D velocity (*v*) and acceleration (*a*) of each bat were calculated numerically by differentiating the smoothed and interpolated position vector. The curvature (ρ) was then calculated using the following equations:

(3) $$a_{n}=a-a_{t}=a-\frac{a\cdot v}{{\left||v|\right|}^{2}}v$$

(4) $$\rho =\frac{\left|\right|a_{n}\left|\right|}{{\left||v|\right|}^{2}}$$

where $a_{n}$ is the normal acceleration and $a_{t}$ is the tangential acceleration with respect to the flight path. The speed was calculated as the norm of the 3D velocity vector at each time step.

After calls had been found and assigned to each bat, they were translated to vectors of binary values. These vectors were down sampled to match the trajectory data. Each index had a value of 1 if there was a call at that time and 0 if there were no calls. These vectors for each pair were then concatenated, similar to the time series data. The final data used for TE analysis were two vectors of position data for the front and rear bat and two vectors with the corresponding call data for the front and rear bats.

The TE analysis was performed on 20 bat pairs together and on two subsets of the data. The partition of the data was based on the time at which the pair flew in front of the cameras and microphone array. Ten of the pairs of bats were recorded in the first 10 min in which the bat colony was leaving the culvert. This will be referred to as set “Begin”. The other 10 pairs of bats were recorded about 45 min later when the majority of the bats had already exited the culvert. This will be referred to as set “End”.

**Figure 8:**
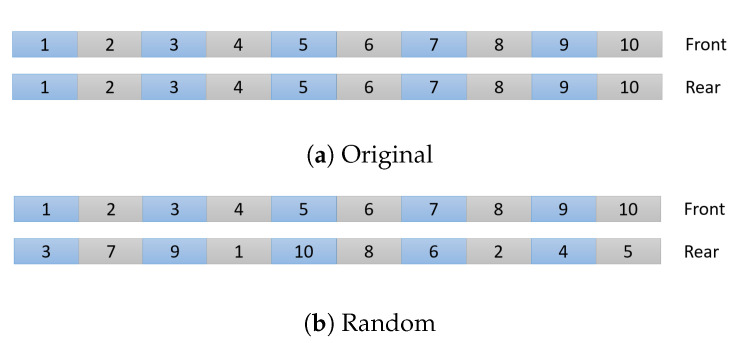
Visual representation of concatenating time series and randomly reassigning pairs.

Since TE analysis requires large sets of data for accurate results, the 1D time series data of each pair was concatenated into two vectors, one containing all the time series data for the front bats and the other all the data for the rear bats. By using this ensemble dataset, we have a single experimental data point. The higher the value of TE, the more information is shared between the data sets. However the value of TE that signifies meaningful information transfer varies for different data sets. To create a baseline for TE values that indicate significant interaction in a pair of bats, the bat pairs in each data set were scrambled. A different rear bat was randomly assigned to each front bat (see Figure 8). Since bats from different pairs were unlikely to have time series of the same length, the longer one of each pair was truncated. The time series for these new pairings were concatenated like before (see Figure 8). TE was calculated for 250 random pairings of bats for each subset of data. The same 250 random pairing were used to calculate the baseline for curvature and speed. If the TE of the real pairing of bats is larger than one standard deviation from the mean of the TE of the random pairings, it is considered to be significant and evidence of information transfer between bat pairs. This approach was selected since it takes into account that the experimental data are a single point rather than a mean of multiple data points, as in for example, a one sample *t*-test.

Transfer entropy was calculated for curvature of trajectories and speed of the bats for all 20 pairs together and each of the subsets. Each of these data types and sets were calculated with calls (multivariate TE) and without calls (univariate TE). Before computing TE, the data were normalized to have zero mean and standard deviation of one. All TE calculations were done using the Java Information Dynamics Toolkit (JIDT) [45]. When calculating TE with a KSG estimator, there is some variance and bias with respect to the true PDF. If the true value is close to zero, the variance in the estimated PDF can give small, negative TE values. Since these negative values mean that there is no information transfer between variables, they are equivalent to zero TE. All calculated TE values that were negative were set equal to zero for analysis.

## 3. Results

**Figure 9:**
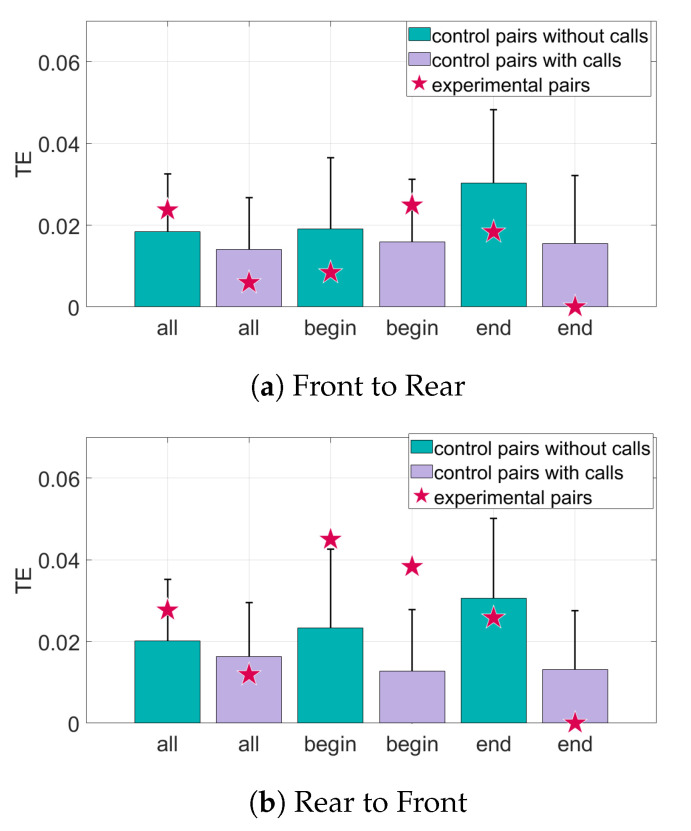
Transfer entropy of curvature of the trajectories for all sets of data. The randomized control pairs are represented by the bar and error plots, where the bar represents the mean of the randomized pairs and the error bar is one standard deviation from the mean. The transfer entropy (TE) of the experimental pairings of bats is represented by the star. The TE of the experimental pairings must be at least one standard deviation from the mean to be considered significant. (**a**) The information transfer from the front bats to the rear bats. (**b**) The information transfer from the rear bats to the front bats.

**Figure 10:**
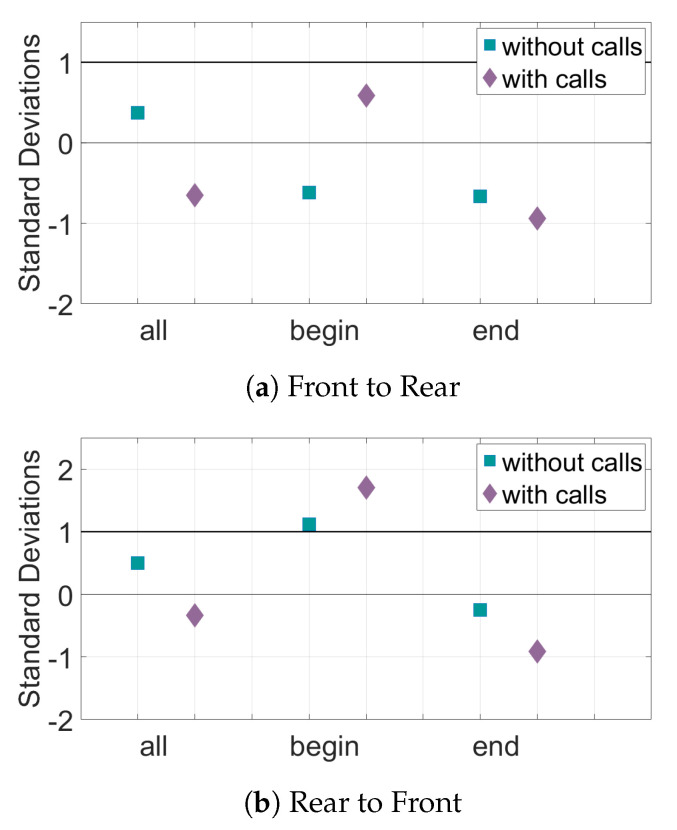
Relative transfer entropy of curvature of the bat trajectories for all sets of data. The horizontal axis represents the different subsets of data, and the vertical axis is the number of standard deviations the experimental pairings are from the mean of the control pairs. Values must be above one standard deviation from the mean of the random pairs (black horizontal line) to be considered significant. (**a**) The information transfer from the front bats to the rear bats. (**b**) The information transfer from the rear bats to the front bats.

The results for the interaction of the bat pairs through the curvature of their trajectories can be seen in Figure 9 and Figure 10. When only position data are considered, the TE values from the rear bats to the front bats of the Begin subset are above the baseline value for significance. However, in the direction front to rear, there is very little information transfer. Adding the data about calls made by the bats increases the TE in the Begin subset in the direction rear to front. However, most of the other subsets in both directions have a decrease in both the TE values (seen in Figure 9) and in distance from mean of the baseline (seen in Figure 10) when call data is included.

**Figure 11:**
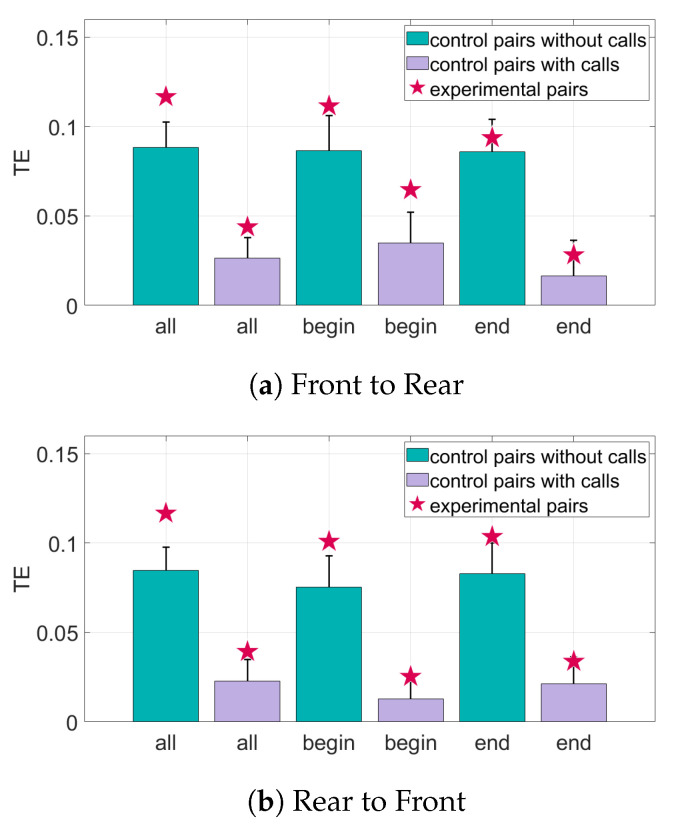
Transfer entropy of the speed of the bats for all sets of data. The randomized control pairs are represented by the bar and error plots, where the bar represents the mean of the randomized pairs and the error bar is one standard deviation from the mean. The TE of the experimental pairings of bats is represented by the star. The TE of the experimental pairings must be at least one standard deviation from the mean to be considered significant. (**a**) The information transfer from the front bats to the rear bats. (**b**) The information transfer from the rear bats to the front bats.

**Figure 12:**
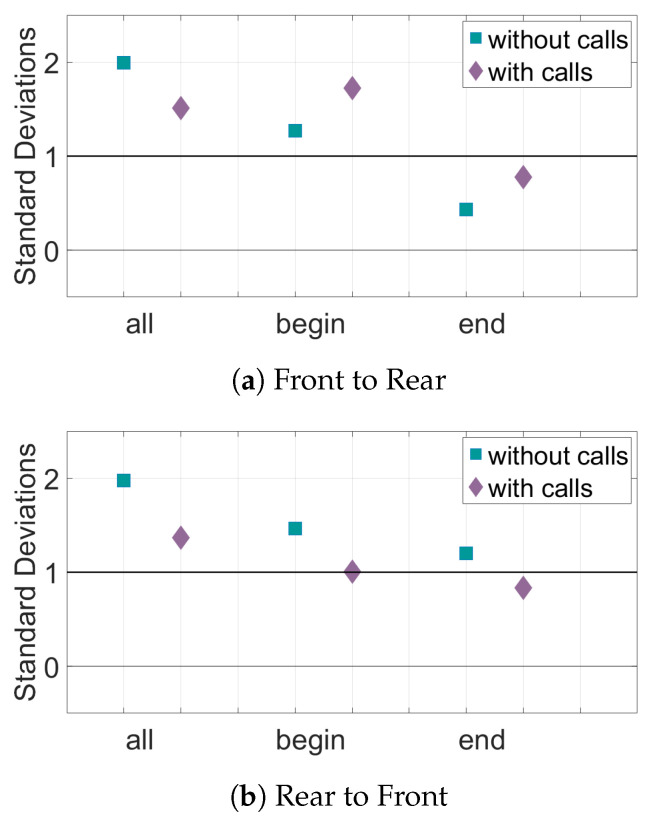
Relative transfer entropy of the speed of the bats for all sets of data. The horizontal axis represents the different subsets of data, and the vertical axis is the number of standard deviations the experimental pairings are from the mean of the control pairs. Values must be above one standard deviation from the mean of the random pairs (black horizontal line) to be considered significant. (**a**) The information transfer from the front bats to the rear bats. (**b**) The information transfer from the rear bats to the front bats.

The results for the interaction of the bat pairs through their speed can be seen in Figure 11 and Figure 12. When looking at the TE of all the pairs of bats, there is significant information transfer both from front to rear and rear to front. Adding data about the calls decreases the TE values but they generally still remain above the baseline for significance. In the Begin subset of pairs, there is also significant information transfer in both directions. In this subset, adding data about calls does not have much effect on the number of standard deviations above the mean of random pairings of the TE, as shown in Figure 12. The End subset only has significant information transfer in the direction rear to front. Adding call data brings the TE value in both directions to just below the baseline value for significance.

## 4. Discussion

For the study performed in this work, the greatest amount of information transfer is seen when the speed of the bats is used for the transfer entropy analysis. This result suggests that, for many of the subsets of bats, one of the bats adjusted its speed due to the speed of the other bat. Analysis of all pairs of bats and the Begin subset show there is information transfer in both directions, meaning there is no leader–follower interaction. In the End subset, there is information transfer from rear to front but not front to rear. However, the difference between the two TE values is small and thus, this may not signify leader–follower interactions. For most subsets, adding data about the echolocation calls does not change the distance of the TE values from the baseline for significance, except in the set of all bats where there is a decrease in information transfer. This indicates that the information flow between the bats’ speeds are not generally changed when the other bat in the pair makes an echolocation call.

**Figure 13:**
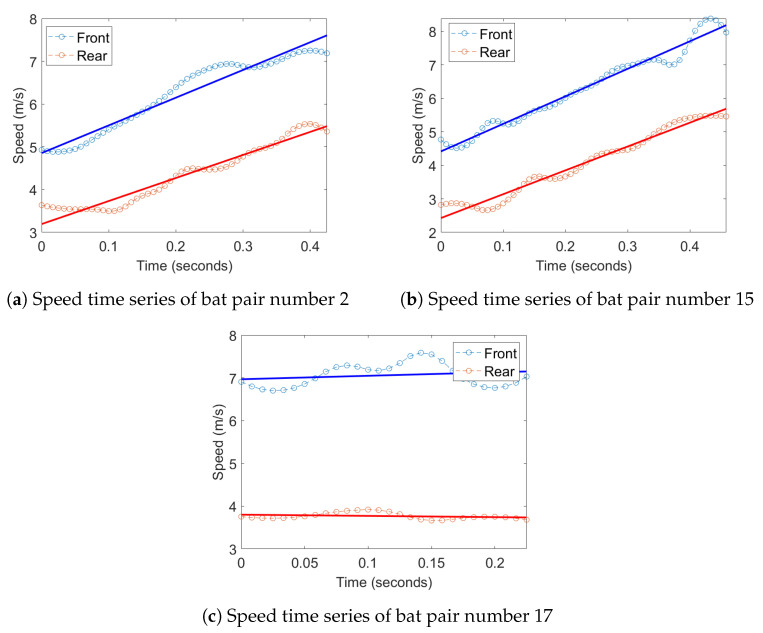
Examples of plots the speed of pairs of bats. A line has been fitted to the data to show the similarity in the slopes of the speed.

Inspection of the time series plots of the speed of the bats shows that the slopes of the line of best fit for the speed plots tend to be similar for the same pair of bats even when the average speed of the two bats varies by more than 2 m/s. This can be seen in example plots in Figure 13. This slope of the speed is the same as the tangential acceleration which is the acceleration of the bats in the direction of travel. This could mean that the pairs of bats are sharing information about their acceleration instead of their speed. Due to the relation between speed and tangential acceleration, the TE analysis finds information transfer in the speed as well. Further analysis needs to be done to confirm this correlation between the tangential acceleration, and could be an area of study for future work on this topic.

The curvature of the trajectories captures more directional information flow between time series representing the turning of the bats. TE analysis of the curvature of the bat trajectories shows evidence of information transfer only in the set of bats that exited the culvert first. In this Begin subset, there is information transfer from the rear bat to the front bat, but no information flow from the front bat to the rear bat. This indicates leadership of the rear bats, which is consistent with the findings in [19]. Including data about calls increases the statistical significance of the information transfer. This suggests that the front bat listens to echolocation calls made by the rear bat and adjusts its curvature according to information learned from the calls.

Comparison of the subsets Begin and End can give some additional insight into the behavior of the bats. There is more information transfer in the Begin subset than the End subset in both the curvature and speed analyses. This could indicate different behavior from the group of bats that leave the roost before the majority of the colony and the group that leaves the roost after the majority of the other bats which may be due to intrinsic characteristics of the bats, like boldness, hunger, or age. An extension of this study could be to record bats over the span of many nights to see if this is a recurring behavior.

Overall, the results from this study are consistent with the result found in a previous study [19], showing that there is evidence of leadership by the rear bat in the curvature of the trajectories. The addition of data from the echolocation calls also supports this finding. This study expanded on the previous study by using a new data set on a different colony of bats, looking at the speed and flight direction of the bats, as well as adding data about the echolocation calls. There are a few areas in which future work could be done to expand on this study. The first would be to record bats at different times of the year or at different times in the same night to see if there are any behavioral changes that can be identified in the information transfer. Another potential study would be to expand the area captured by the cameras. This could give more information on how distance affects the interaction between in a pair of bats. The previous study [19] also looked at how obstacles affected interaction, and this could be studied in further detail by adding data from the echolocation calls made by the bats.

## 5. Conclusions

Based on the results from this study, we propose some theories about the behavior of the bats that were recorded. The increased information transfer in the beginning set of bats compared to the ending set of bats suggests that some characteristics of the first bats to leave the colony were different from the last bats to leave the colony. A possible explanation for this is that the bats who left early were hungry and begin to forage for insects immediately after leaving the culvert, where as the bats who left later might have been less hungry and did not start hunting immediately. The study by Chiu [48] suggests that bats follow each other when capturing insects. Thus, the bats who left the culvert early and immediately started hunting might follow each other more than bats that are flying without searching for food.

Another possible theory is that the age of the bats plays a role in the amount of their interaction. The colony at which these data were collected was a maternity colony. At the time of year the data were collected, the population of bats was made up of adult female bats and nearly fully grown juveniles. It is possible that certain pairings of bats are more likely to share information each other than other pairings. For example, a juvenile might be more likely to follow an adult bat, since the older bat may be better at navigation and foraging. Under this assumption, it is possible that the first bat pairs to leave the culvert were made up of more mixed-age bats that wanted to follow each other than the bat pairs leaving the culvert later.

Overall this study gives insight into the behavior of bats in flight. Beyond the experimental extensions detailed in the discussion, it would be possible to use other information theoretic measures, such as mutual information or other entropies. These measures may highlight different trends in the data that are not revealed by transfer entropy and may offer a more nuanced view of information transfer between the bats.

## References

[1] Parrish J.K., Hamner W.M. (1997). Animal Groups in Three Dimensions: How Species Aggregate.

[2] Krause J., Ruxton G.D. (2002). Living in Groups.

[3] Pilkiewicz K., Lemasson B., Rowland M., Hein A., Sun J., Berdahl A., Mayo M., Moehlis J., Porfiri M., Fernández-Juricic E. (2020). Decoding collective communications using information theory tools. J. R. Soc. Interface.

[4] Lemasson B.H., Anderson J.J., Goodwin R.A. (2009). Collective motion in animal groups from a neurobiological perspective: The adaptive benefits of dynamic sensory loads and selective attention. J. Theor. Biol..

[5] Strömbom D. (2011). Collective motion from local attraction. J. Theor. Biol..

[6] Shannon C.E. (1948). A mathematical theory of communication. Bell Syst. Tech. J..

[7] Stramaglia S., Wu G.R., Pellicoro M., Marinazzo D. (2012). Expanding the transfer entropy to identify information circuits in complex systems. Phys. Rev. E.

[8] Lizier J.T., Heinzle J., Horstmann A., Haynes J.D., Prokopenko M. (2011). Multivariate information-theoretic measures reveal directed information structure and task relevant changes in fMRI connectivity. J. Comput. Neurosci..

[9] Vicente R., Wibral M., Lindner M., Pipa G. (2011). Transfer entropy—a model-free measure of effective connectivity for the neurosciences. J. Comput. Neurosci..

[10] Prokopenko M., Boschetti F., Ryan A.J. (2009). An information-theoretic primer on complexity, self-organization, and emergence. Complexity.

[11] Martinoli A., Easton K., Agassounon W. (2004). Modeling swarm robotic systems: A case study in collaborative distributed manipulation. Int. J. Robot. Res..

[12] Dudek G., Jenkin M.R., Milios E., Wilkes D. (1996). A taxonomy for multi-agent robotics. Auton. Robot..

[13] Runge J., Heitzig J., Petoukhov V., Kurths J. (2012). Escaping the curse of dimensionality in estimating multivariate transfer entropy. Phys. Rev. Lett..

[14] Ver Steeg G., Galstyan A. Information-theoretic measures of influence based on content dynamics. Proceedings of the Sixth ACM International Conference on Web Search and Data Mining.

[15] Bauer T.L., Colbaugh R., Glass K., Schnizlein D. Use of transfer entropy to infer relationships from behavior. Proceedings of the Eighth Annual Cyber Security and Information Intelligence Research Workshop.

[16] Butail S., Mwaffo V., Porfiri M. (2016). Model-free information-theoretic approach to infer leadership in pairs of zebrafish. Phys. Rev. E.

[17] Richardson T.O., Perony N., Tessone C.J., Bousquet C.A., Manser M.B., Schweitzer F. (2013). Dynamical coupling during collective animal motion. arXiv.

[18] Lord W.M., Sun J., Ouellette N.T., Bollt E.M. (2016). Inference of causal information flow in collective animal behavior. IEEE Trans. Mol. Biol. Multi-Scale Commun..

[19] Roy S., Howes K., Müller R., Butail S., Abaid N. (2019). Extracting Interactions between Flying Bat Pairs Using Model-Free Methods. Entropy.

[20] Orange N., Abaid N. (2015). A transfer entropy analysis of leader-follower interactions in flying bats. Eur. Phys. J. Spec. Top..

[21] Butail S., Ladu F., Spinello D., Porfiri M. (2014). Information flow in animal-robot interactions. Entropy.

[22] Nelson M.E., MacIver M.A. (2006). Sensory acquisition in active sensing systems. J. Comp. Physiol. A.

[23] Thomas J.A., Moss C.F., Vater M. (2004). Echolocation in Bats and Dolphins.

[24] Ulanovsky N., Fenton M.B., Tsoar A., Korine C. (2004). Dynamics of jamming avoidance in echolocating bats. Proc. R. Soc. London. Ser. B Biol. Sci..

[25] Betke M., Hirsh D.E., Makris N.C., McCracken G.F., Procopio M., Hristov N.I., Tang S., Bagchi A., Reichard J.D., Horn J.W. (2008). Thermal imaging reveals significantly smaller Brazilian free-tailed bat colonies than previously estimated. J. Mammal..

[26] McFarlane D.A., Keeler R.C., Mizutani H. (1995). Ammonia volatilization in a Mexican bat cave ecosystem. Biogeochemistry.

[27] Bates M.E., Stamper S.A., Simmons J.A. (2008). Jamming avoidance response of big brown bats in target detection. J. Exp. Biol..

[28] Hase K., Miyamoto T., Kobayasi K.I., Hiryu S. (2016). Rapid frequency control of sonar sounds by the FM bat, Miniopterus fuliginosus, in response to spectral overlap. Behav. Process..

[29] Hiryu S., Bates M.E., Simmons J.A., Riquimaroux H. (2010). FM echolocating bats shift frequencies to avoid broadcast–echo ambiguity in clutter. Proc. Natl. Acad. Sci. USA.

[30] Jarvis J., Jackson W., Smotherman M. (2013). Groups of bats improve sonar efficiency through mutual suppression of pulse emissions. Front. Physiol..

[31] Adams A.M., Patricio A., Manohar R., Smotherman M. (2019). Influence of signal direction on sonar interference. Anim. Behav..

[32] Lin Y., Abaid N., Müller R. (2016). Bats adjust their pulse emission rates with swarm size in the field. J. Acoust. Soc. Am..

[33] Chiu C., Xian W., Moss C.F. (2008). Flying in silence: Echolocating bats cease vocalizing to avoid sonar jamming. Proc. Natl. Acad. Sci. USA.

[34] Koay G., Kearns D., Heffner H.E., Heffner R.S. (1998). Passive sound-localization ability of the big brown bat *(Eptesicus Fuscus)*. Hear. Res..

[35] Corcoran A.J., Weller T.J. (2018). Inconspicuous echolocation in hoary bats *(Lasiurus Cinereus)*. Proc. R. Soc. B Biol. Sci..

[36] Ristau R. (2017). Analysis Finds 37 of Nearly 500 Bridges in Region Structurally Deficient. https://www.heraldcourier.com/news/analysis-finds-of-nearly-bridges-in-region-structurally-deficient/article_fa077501-077d-5e91-8f8e-9be20b967ecf.html.

[37] Zhang Z. (2000). A flexible new technique for camera calibration. IEEE Trans. Pattern Anal. Mach. Intell..

[38] MathWorks (2020). Help Center: What Is Camera Calibration. https://www.mathworks.com/help/vision/ug/camera-calibration.html.

[39] Hedrick T.L. (2008). Software techniques for two-and three-dimensional kinematic measurements of biological and biomimetic systems. Bioinspir. Biomimetics.

[40] Theriault D.H., Fuller N.W., Jackson B.E., Bluhm E., Evangelista D., Wu Z., Betke M., Hedrick T.L. (2014). A protocol and calibration method for accurate multi-camera field videography. J. Exp. Biol..

[41] Lourakis M.I., Argyros A.A. (2009). SBA: A software package for generic sparse bundle adjustment. ACM Trans. Math. Softw. (TOMS).

[42] MathWorks (2020). Help Center: Smooth. https://www.mathworks.com/help/curvefit/smooth.html.

[43] Yovel Y. (2020). Batalef—Audio Analysis Software for Animal Research. www.yossiyovel.com.

[44] Schreiber T. (2000). Measuring information transfer. Phys. Rev. Lett..

[45] Lizier J.T. (2014). JIDT: An information-theoretic toolkit for studying the dynamics of complex systems. Front. Robot. AI.

[46] Kraskov A., Stögbauer H., Grassberger P. (2004). Estimating mutual information. Phys. Rev. E.

[47] Gómez-Herrero G., Wu W., Rutanen K., Soriano M.C., Pipa G., Vicente R. (2015). Assessing coupling dynamics from an ensemble of time series. Entropy.

[48] Chiu C., Reddy P.V., Xian W., Krishnaprasad P.S., Moss C.F. (2010). Effects of competitive prey capture on flight behavior and sonar beam pattern in paired big brown bats, Eptesicus fuscus. J. Exp. Biol..

